# Environmental Instability as a Motor for Dispersal: A Case Study from a Growing Population of Glossy Ibis

**DOI:** 10.1371/journal.pone.0082983

**Published:** 2013-12-20

**Authors:** Simone Santoro, Andy John Green, Jordi Figuerola

**Affiliations:** Department of Wetland Ecology, Estación Biológica de Doñana, Consejo Superior de Investigaciones Científicas (CSIC), Sevilla, Spain; University of Missouri-Columbia, United States of America

## Abstract

Dispersal is a life-history trait directly affecting population dynamics and species range shifts and thus playing a prominent role in the response to climate change. Nonetheless, the relationship between extreme climatic events and dispersal has received little attention in birds. Here we focused on climatic, demographic and individual factors affecting the dispersal propensity of a major glossy ibis population. We performed a capture-resighting analysis on individuals born and observed at Doñana (South-West Spain) over fourteen years. We applied a multiple analytical approach to show that single-site capture-resighting estimates were a reliable index of dispersal propensity from the area. We focused on the emigration of Doñana-born individuals sporadically (transients) and regularly (residents) frequenting their natal area. Droughts during two out of 14 study years caused higher apparent dispersal rates, explaining most of the annual variation in these rates. The age structure of Doñana-born individuals resighted simultaneously locally and in Morocco in one week over the 2010 autumn confirmed that the 2005 drought boosted permanent emigration. As numbers increased steadily during non-drought years since the formation of the colony in 1996 to several thousand pairs, philopatry increased gradually, while transients probability appeared to be related to average breeding success. Age, sex, density, quality of foraging habitat and breeding success in the previous season were not found to directly affect apparent dispersal. Nonetheless, autumn sex ratio gradually switched from male (≈0.68) to female-skewed (≈0.44) by the end of the study period, suggesting that males and females respond differently to high densities reached in recent years. This study demonstrates the importance of extreme climatic events as a powerful motor for spread of species in expansion. Also, it suggests different factors drive emigration of individuals according to their amount of experience in the area (e.g. transients vs residents).

## Introduction

Dispersal propensity has major consequences for population structure and dynamics, behavioral ecology and conservation of birds and other organisms [1]. Dispersal can be an adaptive response to environmental changes, or social condition [2]. Dispersal also allows an immediate response to unfavorable extreme events such as hurricanes, floods and droughts and is a key element of response to climate change [3], [4]. Although research on the effect of extreme events on dispersal dynamics of birds is badly needed [5], this topic has still received little attention from ecologists [6].

Dispersal can be seen as a three-stage movement with emigration, transfer and immigration phases [2]. At the individual level, factors such as status (e.g. age or sex), condition (e.g. body condition, social status), or personality may act as proximate causes of dispersal. For example, juveniles may disperse more than adults because they may have less to lose by moving away [1]. Aggressiveness [7] or individual condition may affect dispersal propensity by changing the ability to compete with conspecifics [8]. At the environmental level, dispersal may be affected by the intrinsic quality of the habitat or by variation of patch quality depending on local resource competition. However, dispersal responses to a given causal factor may vary. For example, dispersal can increase at higher densities as a result of increased competition [9], but it can also decrease at higher density [10], [11] owing to conspecific-attraction [12], lower predation risk or Allee effects. Positive and negative density-dependent effects on dispersal can even co-occur in the same population [11], [13] demonstrating how the decision to disperse may be affected by other factors. Hence, for a specific population, a positive or negative density effect may depend on being above or below a certain threshold. In newly established bird populations, this can be tested by studying the relationship between time since colonization and dispersal propensity. Thus, Duckworth and Badyaev [7] found that philopatry increased with time elapsed since colonization in a passerine bird.

The glossy ibis (*Plegadis falcinellus*) is one of the six most widely distributed landbird species [14], and this is probably related to great dispersal ability. It has been suggested that the new World was recently colonized by individuals of this species crossing the Atlantic [15]. Different observations of Doñana born individuals in Barbados (one in autumn 2010), Bermudas (one in autumn 2013) and Trinidad (one in summer 2008) indicate this species can disperse crossing the Atlantic, flying almost 6000 Km away from natal sites (authors unpublished data). The glossy ibis is a migratory and dispersive species with nomadic elements [16], but there have been no previous detailed studies of its dispersal behaviour. At Doñana, seven glossy ibis breeding pairs established in 1996. Since then their number increased quickly to several thousand pairs [17] and Doñana now holds the most important breeding [18] and probably wintering population in western Europe.

The main aim of this study was to test hypotheses about how environmental and individual factors influence the dispersal propensity of a population in expansion, the glossy ibis in Doñana. In the classical mark-recapture multi-site framework [19] longitudinal data from well monitored populations at several locations are used to test hypotheses about the dispersal process (e.g. [20]) of a metapopulation [21]. In the case of glossy ibis, the Doñana and Camargue populations are the only large, well-monitored population, and data from other areas in Europe are sparse. Here, we tested hypotheses about the leaving stage of the dispersal process using a combination of uni-site multievent capture-recapture models [22] on resighting data, and comparison of resightings from within and outside Doñana. We used autumn-to-autumn data because at that time abundant food was regularly available in Doñana rice-fields, and birds were easier to approach for ring reading. We tested the influence of age and sex as individual factors influencing the dispersal propensity. Furthermore, since dispersal dynamics are strongly related to conditions and individual experience during the breeding season [1], we focused on how environmental conditions during the breeding season may affect the dispersal rate between two consecutive autumns. In particular, we tested how dispersal propensity changes in response to the dramatic changes in population size, annual variation in breeding success and food supplies, and to drought years that make Doñana unsuitable for breeding.

## Materials and Methods

### Study Area and Individual Data

Doñana is an extremely important wintering and breeding area for waterbirds [23]. Doñana National park contains about 27000 ha of natural marshes, which provide the main breeding area for glossy ibis [24]. It is surrounded by ca. 36000 ha of rice-fields that provide abundant food to the ibis after harvest [25]. It is characterized by a Mediterranean climate with mild, wet winters and hot, dry summers. Springs in 1999 and 2005 were characterized by extreme local droughts with the flooded surface of natural marshes in June below the 10th percentile of a distribution for the period between 1981 (when remote sensing data were first available for the area) and 2011. This prevented glossy ibis and most waterbirds from breeding at Doñana in these two years. Hereafter we refer to “dry” and “wet” years to distinguish autumns (October–December, the months from which capture - recapture data were obtained) that were followed by spring drought. Therefore, 1998 and 2004 were defined as dry and all the others as wet years. Capture-recapture data in the present study consisted uniquely of resightings. However, for the sake of simplicity, we use the traditional term “trap-happiness” to refer to repeated resightings of the same individual, even though no physical trapping is involved.

Since the establishment of a breeding population of glossy ibis at Doñana (Southwest Spain) in 1996, more than 15000 chicks have been ringed and marked with a darvic ring with an alphanumeric 3–4 digits individual code. These rings may be read with the aid of a telescope from up to 100 meters. Almost 4000 chicks were molecularly sexed (following [26]) and a partially overlapping set of more than 10000 were visually sexed based on tarsus shape [27].

Ringing and blood sampling was approved by Doñana National and Natural Park, Regional Andalusian Government (Consejería de Medio Ambiente), and the Animal Bioethics Committee from Doñana Biological Station. Ringing procedures were approved by the Spanish Ministry of Environment (according to Law 8/2003).

### Goodness of Fit

We tested the Goodness of Fit (GOF) of the simple Cormack-Jolly-Seber model separately for birds first resighted in autumn as juveniles or as adults (>1 year old). This approach is conservative as it tests the fit of a more general model with stronger assumptions (i.e. no site or sex differences). The GOF was tested with U-CARE 2.3.3 [28]. The variance inflation factor (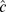) was derived from the global test as 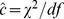, where 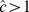 indicates over-dispersion. The global test was statistically significant (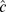 = 2 (183.43/92); *P*<0.001) detecting both a transience effect (of both juveniles and adults) and “trap-happiness” (of juveniles). By “transience effect” we refer to presence of individuals showing up only once in the study area after fledging. Thus, a transience effect can be caused by (*i*) presence of individuals passing through the study area with no chance of returning and/or, (*ii*) by higher mortality rate just after the first resighting. Tavecchia et al. [29], studying Audouin’s gull (*Larus audouinii*), showed that many transients dispersed permanently to other areas. On the other hand, “Trap-happiness” of birds first-resighted as juveniles may reflect a lower propensity to temporary emigration in juveniles or a higher fidelity during the first years of life to habitats, like Doñana ricefields, where most of the observations were made. When first encounters were removed, the global test was no longer significant [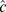 <1 (62.98/68); P = 0.65; File S1 ]. As a consequence, we built models by considering a specific parameter type accounting for transience probabilities (we called it “Transience”) and included a time-since-marking effect in the global model, which assigned different probabilities for the first re-encounter of juveniles.

### Capture-resighting Modeling

We pooled data from October to December, the months in which the majority of observations of marked birds were made. This resulted in 5037 resightings made at Doñana of 3440 individuals observed from 1998 to 2011 (1996 and 1997 were excluded because there were only a handful of marked individuals and they were not sexed). Only sex scores from ringers expert in handling the species were considered in the analyses: of 3440 chicks, 669 (19.4%) were both molecularly and visually sexed, 326 only molecularly sexed, and 1664 only visually sexed. Consequently, we knew with certainty the sex of a portion of individuals, whereas most individuals were visually sexed and therefore subject to error. This uncertainty in sex determination was incorporated into capture-resighting multievent models ([22], File S2) that distinguish what is observed in the field (events) from the underlying biological states.

In this study we considered three states: (*i*) live female, (*ii*) live male, (*iii*) dead or transient (i.e. an individual showing up only once); and four events with the corresponding number used in the data set: (*i*) not seen – “0”, (*ii*) seen and visually sexed at birth as female – “1”, (*iii*) seen and visually sexed at birth as male – “2”, and (*iv*) seen but with sex not determined molecularly nor visually – “3”. Multievent models include three parameter types: (*i*) Initial State probabilities, (*ii*) Transition probabilities among states, and (*iii*) Event probabilities conditional on the underlying states.

We set Initial State to be the probability that a bird resighted for the first time was a male. This parameter can be related to the sex ratio of the population at each session if, as in our case, sex had no effect on resighting probabilities and the sampling scheme was not changed over the study period [30], [31]. For simplicity, hereon we will often refer to it as “sex ratio”.

The Transition between states referred to the probability of so-called “local mortality” that here we call “apparent dispersal”, i.e. the probability that an individual will die or permanently emigrate between *t* and *t*+1. This parameter type was broken down into two steps represented by “Transience” and “Residence” matrices. The Transience matrix is related to the probability an individual observed for the first time had of death or permanent emigration before the next autumn, for analogy with apparent dispersal we call it “apparent transience”. Hence, the apparent transience probability after the first interval since the first observation is, by definition, zero (details in File S2 and File S5). By contrast, the Residence matrix relates to the apparent dispersal probability of an individual conditional on it being alive and not being a transient after its first interval. Thus, on the one hand the Transience matrix allows estimation of the probability of being a transient (or dying in the first interval). On the other hand, the Residence matrix allows estimation of apparent dispersal of philopatric individuals (residents). For the sake of convenience, hereon we refer to both these parameter types (Transience and Residence) together in combination as unspecified “apparent dispersal” to differentiate it from “apparent dispersal of residents”.

**Figure 1:**
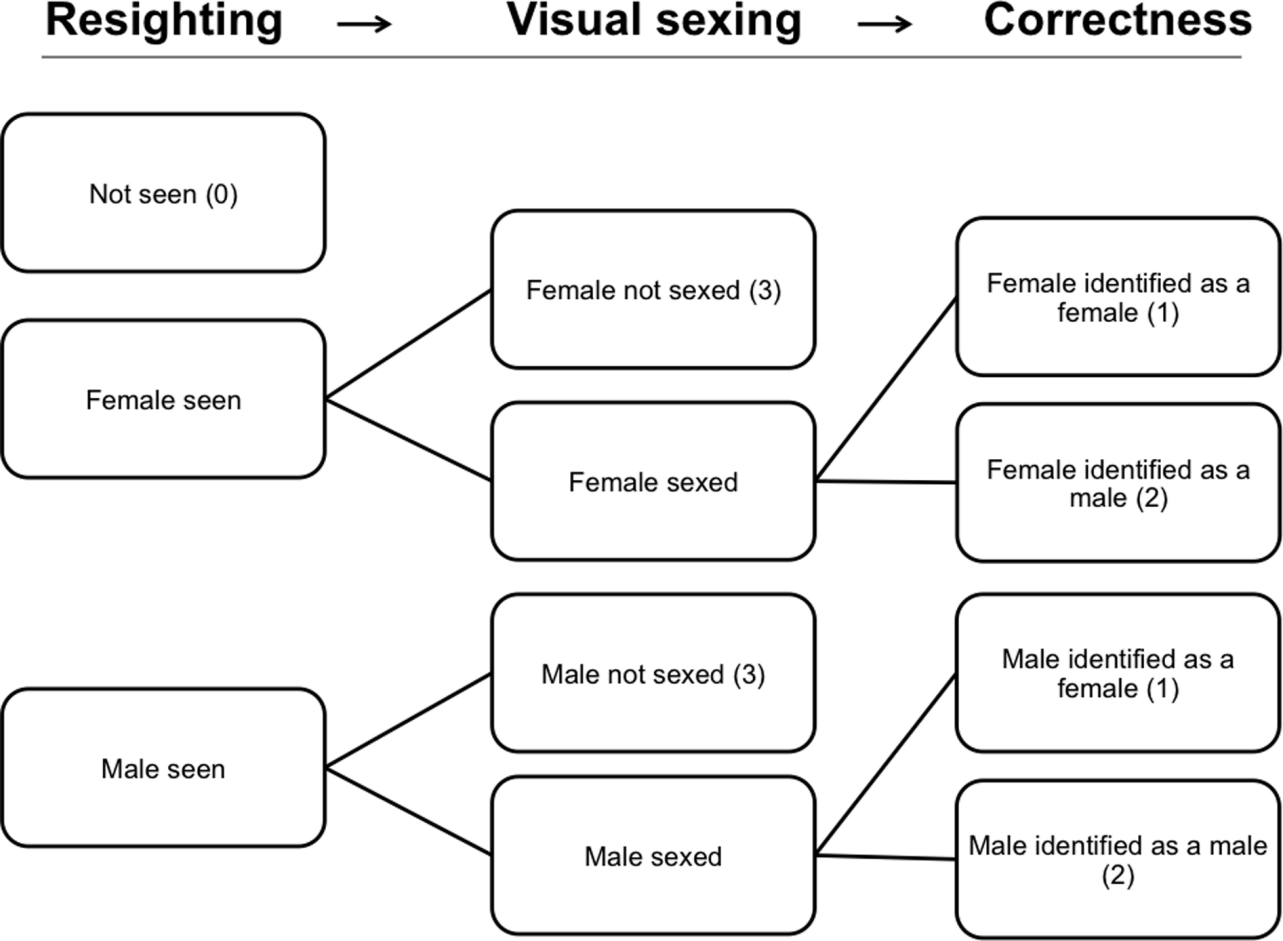
Events and underlying states. The observation process consists of three steps, each one conditional on the event in the previous step. The codes for each event as they appear in the raw data for encounter histories are given in parentheses.

Event probabilities were broken down into three steps (Figure 1): (*i*) “Resighting”, i.e. the probability of being “resighted”, (*ii*) “Visual Sexing”, the probability of being visually sexed when first captured at the colony, and (*iii*) “Correctness”, the probability of being correctly sexed visually as a female or a male.

We tested the effect of five external covariates on apparent dispersal: (*i*) droughts, (*ii*) breeding success (average n. of fledgings) during the previous and current reproductive seasons ([32], authors unpublished data), (*iii*) population density proxied by breeding population size (see [17]) in the previous spring and (*iv*) food supplies proxied by the flooded surface in the Guadalquivir marshes in the current spring (June, measured from Landsat images, see [33]). We checked the effects on apparent dispersal of different factors alone, and since drought explained most variation in this parameter, we also tested the effect of the other factors separately on the set of non-drought years. At the individual level, we also tested the effects of age and sex on apparent dispersal.

#### Model selection

Models were built and fitted to the data using program E-SURGE 1.8.5 [34]. We used the sample-size adjusted Akaike’s Information Criterion (AICc) as the model selection criterion [35]. Our model selection procedure was analogous to that proposed by Grosbois and Tavecchia [36]. It aimed to: (*i*) minimize the bias due to the modeling order followed during model selection and, (*ii*) avoid the problematic large number of models arising from considering all the possible combinations of effects and parameter types. In particular, we considered two blocks of parameter types (one for Event, another for Initial State and Transition) and applied a model selection procedure consisting of two different series for each block. In the first series, we modeled each parameter by keeping the others as generalized as possible, in the second we ran models arising from the combinations of the best model structures found in the first series for each parameter (see File S3 for details of the model selection procedure). Hence, we relied mainly on models from series one to test hypotheses, and used models from series two to average parameter estimates [35]. The detailed results from series two are presented in supplementary material (Tables S1, Table S2 and Table S3 in File S4). See File S5 for details of implementation in E-SURGE.

We used the ANODEV procedure [37] to test the hypotheses of the covariate models. This test compares the deviances of the null, the full time-dependent, and the environmental covariate models to calculate a statistic that follows a Fisher-Snedecor distribution (see [20] for details). The percentage of variation explained by each tested covariate was computed from the deviance of the models, as by Sanz-Aguilar et al. [20]. Spurious effects can be detected when both the parameter to be estimated and the covariate being tested show a linear trend through time ([38], see [39] for an example with density-dependence). Hence, in cases where we detected a trend on a biological parameter, we used the de-trended [40] breeding population size (known to be linearly increasing, [17]) to test for density-dependence.

### Apparent Dispersal: A Good Proxy for Real Dispersal?

As mentioned above, apparent dispersal variation might be due to both true mortality and permanent emigration from the area. Even though we could not ascertain which part of this parameter was due to true mortality and which to permanent emigration, we aimed to test our working-assumption that variation in true dispersal over time was related to variation in “apparent dispersal” over time. To this purpose, we used several different approaches (see next three sections).

#### Resightings outside doñana versus apparent dispersal at doñana

First, we tested for a relationship between the proportion of Doñana glossy ibises resighted outside Doñana the following autumn session and “apparent dispersal”. If these parameters (i.e. apparent transience combined with apparent dispersal of residents) varied along with dispersal propensity we would expect to find support for this model. Given that observations outside Doñana are mainly made by amateur bird-watchers, we assumed resighting effort was roughly constant throughout the study period (we excluded observations made by our team in Morocco in 2010). Nonetheless, the number of observations outside Doñana might depend on changes in population size and not on real changes in dispersal probability. Hence, assuming resighting probability outside Doñana was constant, we used the ratio between the number of resightings outside Doñana and an index of population size (*N_t+1_*) for each *t*+1 session as a covariate of resighting rate, computed as:
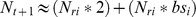
where *N_ri_* and *bs_i_* refer respectively to the number of breeding pairs in Doñana and their breeding success (number of fledgings per pair) at the reproductive season *i* between *t*−>*t*+1. Then, we tested this effect on apparent dispersal via the models in block two series one (File S3) and tested the significance of the effect according to the ANODEV procedure.

#### Doñana and morocco autumn populations

To obtain estimates of dispersal independent of mortality, two teams simultaneously collected resightings over five days (12/11/2010–16/11/2010) in Doñana and Morocco (Larache and Souss-Massa) wetlands. Both teams were well trained in reading darvic rings from distance and used the same equipment (20X–60X zoom telescopes). Thus, we compared the proportion of individuals from each cohort and the total sex ratio of individuals resighted in Doñana and Morocco.

#### Dispersal range in autumn

We described the dispersal range of Doñana-born glossy ibises based on observations from elsewhere. We used only resightings more than 100 Km away from Doñana and considered the same time intervals (October – December) used in the capture-resighting analyses, plus January since this is the month when extensive efforts are made to count waterbirds during the International Waterbird Census (IWC) carried out in most range countries. Also, we tested for differences in the proportion of males and females seen at Doñana or elsewhere. Due to sparseness of data, we did not test for sex ratio differences on annual basis but considered only: (*i*) all the autumns pooled together, (*ii*) autumn 2005 (immediately after the last drought) and, (*iii*) from autumn 2005 onwards (when dispersal range appeared to increase).

## Results

### Capture-resighting Analyses

#### External factors affecting apparent dispersal

**Figure 2:**
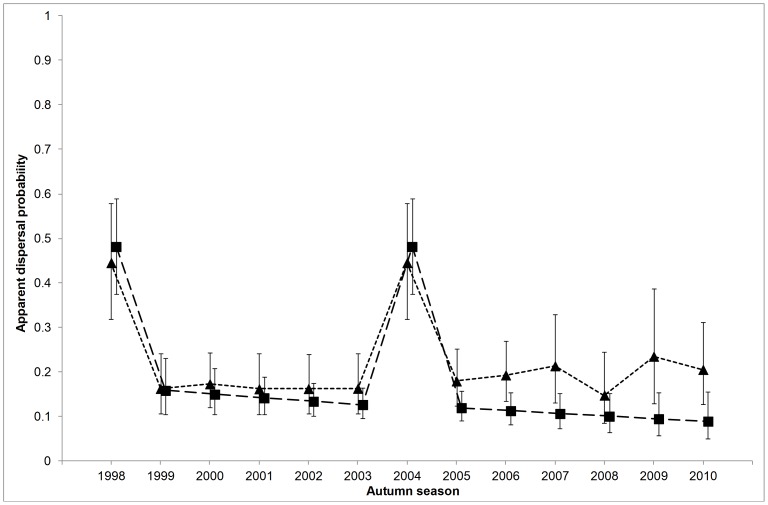
Model averaged estimates of apparent dispersal rates throughout the study period. Apparent dispersal refers to the probability of dying or permanently emigrating from Doñana. Apparent transience (triangles, short-dashed line) was much more pronounced in dry years (1998 and 2004), without any clear trend for other years. Apparent dispersal of residents (squares, long-dashed line) followed a linear decreasing trend over the study period. Estimates and 95%CI are from the model with the lowest AICc (model 22–2 in File S4: Table S1 in File S4).

Apparent dispersal rates for the two time-intervals comprising a drought were much higher than for the other years. Based on the point estimates ratios, on average apparent transience had 2.5-times higher in dry years whereas the apparent dispersal of residents was 4.0 times higher (Figure 2). Probabilities of apparent transience and apparent dispersal of residents were similar in dry years (0.44, SE = 0.07; 0.49, SE = 0.05, respectively).

For Transience modeling, the model accounting for droughts alone was the most supported and explained 60% of time variation over time (*F*_1,11_ = 6.65, *P* = 0.026; model 21–6 in Table 1). Nonetheless, a model accounting for effects of both drought years and breeding success in wet years (*bs*) was selected as the most parsimonious (model 22–2: Table S1 in File S4) in model selection series two (see File S3). Averaged model estimates for apparent transience were computed from this final model selection step (Table S1 in File S4), indicating that the lowest estimate [0.15, (SE = 0.04)] coincided with the highest productivity (in 2009, 2.7 fledgings per pair), and the highest estimate [0.23, (SE = 0.07)] coincided with the lowest productivity (in 2010, 0.87 fledgings per pair) (Figure 2).

**Table 1:** Multievent modeling of apparent transience (Transience matrix) of glossy ibises related to external covariates.

Model	Transients dispersal	*np*	Dev	AICc	ΔAICc	*wi*	*F*_1,11_/*F*_2,10_	*P*-value	*F*_1,9_	P-value	*R* ^2^_tot_	*R* ^2^_wet_
21-6	Dry years	68	16028.50	16166.39	0.00	0.30	6.65	0.026			0.60	
21-15	Dry years, *bs* on wet years	69	16027.45	16167.39	1.00	0.18	3.22	0.080	0.94	0.360	0.64	0.10
21-17	Dry years, *fgm* on wet years	69	16028.11	16168.06	1.67	0.13	3.10	0.090	0.35	0.570	0.62	0.04
21-14	Dry years, *dens* on wet years	69	16028.26	16168.20	1.81	0.12	3.07	0.090	0.22	0.650	0.61	0.02
21-13	Dry years, *T* on wet years	69	16028.45	16168.39	2.00	0.11	3.03	0.090	0.05	0.830	0.61	0.01
21-16	Dry years *bs_t-1_* on wet years	69	16028.48	16168.43	2.04	0.11	3.03	0.090	0.02	0.890	0.61	0.00
21-9	*bs*	68	16032.17	16170.05	3.66	0.05	5.11	0.046			0.46	
21-gm	*T*	78	16018.10	16176.58	10.19	0.00						
21-12	Dry years, *t* on wet years	78	16018.42	16176.90	10.51	0.00						
21-11	*fgm*	68	16041.33	16179.22	12.83	0.00	1.28	0.280			0.12	
21-5	*Constant*	67	16044.39	16180.22	13.83	0.00						
21-10	*bs0*	68	16043.98	16181.87	15.48	0.00	0.17	0.690			0.02	
21-8	*dens*	68	16044.11	16182.00	15.61	0.00	0.12	0.740			0.01	
21-7	*T*	68	16044.38	16182.27	15.88	0.00	0.00	0.960			0.00	

Model notation: np, number of estimable parameters; Dev, relative deviance; AICc, Akaike information criterion corrected for small sample size; ΔAICc, the AICc difference of the current model with respect to the lowest AICc value; w_i_, Akaike’s weight, *F*_1,11_/*F*_2,10_, F-statistic computed for the whole period of study, number of d.f. depends on the current model; *F*_1,9_, F-statistic computed for the wet years; *R* ^2^_tot_, current model percentage of variation explained over the whole study period; *R* ^2^_wet_, current model percentage of variation explained over the wet years; *bs*, breeding success (n° of fledgings per pair) for the reproductive season between autumn seasons *bs_t-1_*, breeding success in the last reproductive season; *dens*, population size in the last breeding season; *fgm*, flooded surface of natural marshes in Doñana National Park in June; *T*, linear trend; *t*, unspecific time variation. All the models were run with unspecific time variation on Initial State and Residence and the best ranked structure for the resighting parameter types (Event).

The most supported model for Residence modeling (model 21–26 in Table 2) suggested that apparent dispersal of residents was affected by droughts and had a negative linear trend over wet years. The model explained 85% of temporal variation over the whole study period (*F*_2,10_ = 4.27, *P*<0.046). The linear trend effect was marginally significant (*F*_1,9_ = 4.37, *P* = 0.066) explaining 49% of apparent dispersal variation in wet years, whose model averaged estimates (Table S1 in File S4) linearly decreased from 0.16 (SE = 0.03) to 0.09 (SE = 0.03) (Figure 2).

**Table 2:** Multievent modeling of apparent dispersal of residents (Residence matrix) of Doñana glossy ibises related to external covariates.

Model	Transients dispersal	*np*	Dev	AICc	ΔAICc	*wi*	*F*_1,11_/*F*_2,10_	*P*-value	*F*_1,9_	P-value	*R* ^2^_tot_	*R* ^2^_wet_
21–26	Dry years, *T* on wet years	70	16022.96	16164.96	0.00	0.54	4.27	0.046	4.37	0.066	0.85	0.49
21–19	Dry years	69	16027.46	16167.40	2.45	0.16	7.90	0.017			0.72	
21–28	Dry years, *bs* on wet years	70	16026.50	16168.50	3.54	0.09	3.73	0.062	0.93	0.430	0.75	0.10
21–27	Dry years, *dens* on wet years	70	16026.50	16168.50	3.55	0.09	3.73	0.062	0.93	0.360	0.75	0.10
21–30	Dry years, *fgm* on wet years	70	16027.18	16169.18	4.22	0.06	3.63	0.065	0.27	0.660	0.73	0.03
21–29	Dry years *bs0* on wet years	70	16027.45	16169.46	4.50	0.06	3.59	0.067	0.01	0.920	0.72	0.00
21-gm	*T*	78	16018.10	16176.58	11.63	0.00						
21–25	Dry years, *t* on wet years	78	16018.18	16176.67	11.71	0.00						
21–22	*bs*	69	16039.72	16179.66	14.71	0.00	3.83	0.076			0.35	
21–23	*bs0*	69	16048.29	16188.23	23.27	0.00	0.99	0.340			0.09	
21–18	*Constant*	68	16051.28	16189.17	24.21	0.00						
21–24	*fgm*	69	16049.70	16189.65	24.69	0.00	0.52	0.490			0.05	
21–21	*dens*	69	16050.28	16190.22	25.26	0.00	0.33	0.580			0.03	
21–20	*T*	69	16051.28	16191.22	26.26	0.00	0.00	0.970			0.00	

Model notation: as in the Table 1. All the models were run with unspecific time variation on Initial State and Transience and best ranked structure for the resighting parameter types (Event).

In wet years, apparent transience and apparent dispersal of residents were very similar at the beginning of the study period. However, in later years the probability of apparent dispersal of residents decreased consistently, whereas the probability of apparent transience did not show any time-trend throughout the study period (Figure 2).

#### Age and sex differences in apparent dispersal

Apparently, juveniles had slightly higher apparent transience probabilities than adults [model 22 in File S4: Table S2 in File S4; slope on logit scale = −0.53 (SE = 0.34)]. Nonetheless, 95% confidence intervals of juveniles and adults overlapped (Figure S1 in File S6) and a model without an age effect was also plausible (model 22 *vs.* model 22-2: Table S2 in File S4; ΔAICc = 0.31). Sex was not very influential on apparent dispersal (Table S2 in File S4). Females had slightly higher apparent dispersal probabilities [Transience, model 23, slope on logit scale = −0.29 (SE = 0.27); Residence, model 25, slope on logit scale = −0.11 (SE = 0.22)] but their 95% confidence intervals overlapped.

#### Sex ratio

**Figure 3:**
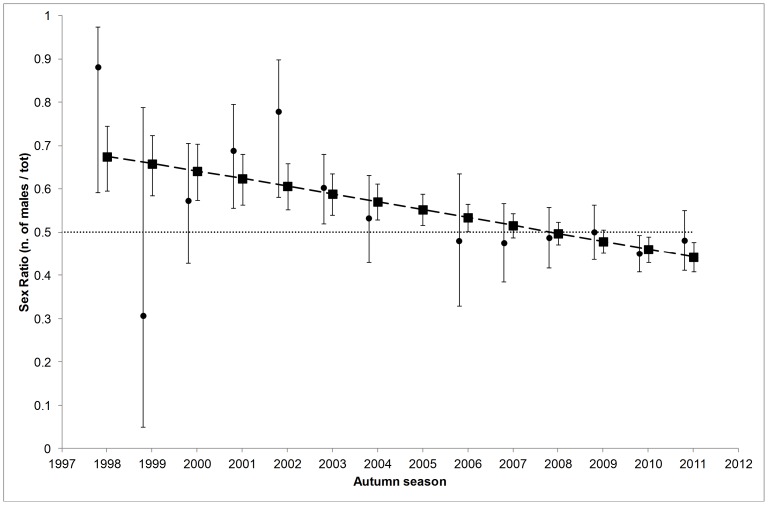
Sex ratio (no. of males over total) of Doñana-born glossy ibises observed in Doñana in autumn. According to the model with lowest AICc (22-2 in Table S1 in File S4), Initial State estimates (95% CI) switched from a majority of males to a majority of females at the end of the study period (squares, long-dashed line). Circles are estimates (95%CI) from a model with unconstrained time variation (there was no estimate for 2005 due to scarce data). The dotted horizontal line indicates a balanced sex ratio.

According to Initial State parameter estimates, autumn sex ratio (no. of males/total) at Doñana showed a significant decreasing trend over time (model 21-3 in Table 3; *F*_1,12_ = 6.42, *P* = 0.026). Density and droughts had no effect on sex ratio. While at the beginning of the study there was a majority of males (in 1998 0.67, SE = 0.04), by the end there was a majority of females (in 2011 0.44, SE = 0.02) (Figure 3).

**Table 3:** Multievent modeling of Doñana autumn sex ratio (Initial State parameter) of native glossy ibises related to external covariates.

Model	Transients dispersal	*np*	Dev	AICc	ΔAICc	*wi*	*F*_1,11_/*F*_2,10_	*P*-value	*R* ^2^_tot_
21-3	*T*	66	16033.44	16167.22	0.00	1	6.42	0.026	0.54
21-gm	*t*	78	16018.10	16176.58	9.37	0.00			
21-1	*Constant*	65	16051.09	16182.82	15.60	0.00			
21-2	Dry years	66	16049.88	16183.66	16.44	0.00	0.44	0.520	0.04
21-4	*dens*	66	16050.81	16184.59	17.38	0.00	0.10	0.760	0.01

Model notation: as in the Table 1 legend. All the models were run with unspecific time variation on Transience and Residence and the best ranked structure for the resighting parameter types (Event).

#### Resightings and visual sexing

Resighting probabilities varied among years, showing a general decrease over time probably due to the increase in the number of marked birds alive (Figure S2 in File S6). On average, female chicks were slightly more likely to be visually sexed than males. Both the probability to be visually sexed and its reliability as a sexing method depended on the cohort, with accuracy ranging from 0.80 (SE = 0.03) to 0.99 (SE = 0.005) (Figures S3 and S4 in File S6).

### Relationship between Real Dispersal and Apparent Dispersal

#### Resightings elsewhere versus apparent dispersal at doñana

Apparent transience was positively related to the proportion of resightings outside Doñana corrected for the population size, but this was not the case for apparent dispersal of residents (Transience: *F*_1,11_ = 6.4, *P* = 0.028; Residence: *F*_1,11_ = 0.83, *P* = 0.38).

#### Doñana and morocco autumn populations

Between 12th and 16th November 2010, 417 Doñana-born individuals were resighted in Doñana and 416 in Morocco. The same cohorts (from 2000 to 2010 except 2005) were represented in both areas (Table S3 in File S4).

Of individuals born in 2004, just before the last Doñana drought, 58 were resighted in Morocco and only 7 in Doñana (Chi-square test 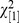 = 35.8, *P*<0.01). A higher than expected number of individuals born in 2003 were also recorded in Morocco (Chi-square test 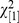 = 8.3, *P*<0.01) while the opposite was found for the 2010 cohort which was relatively more represented in Doñana (Chi-square test 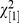 = 8.3, *P*<0.01) indicating a higher presence of very young individuals (8–10 months old) at the natal site (Table S3 in File S4). The same proportion of males and females was found in Doñana and Morocco (Chi-square test 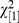 = 0, *P* = 1; Doñana: n = 87; Morocco: n = 110).

#### Dispersal range in autumn

**Figure 4:**
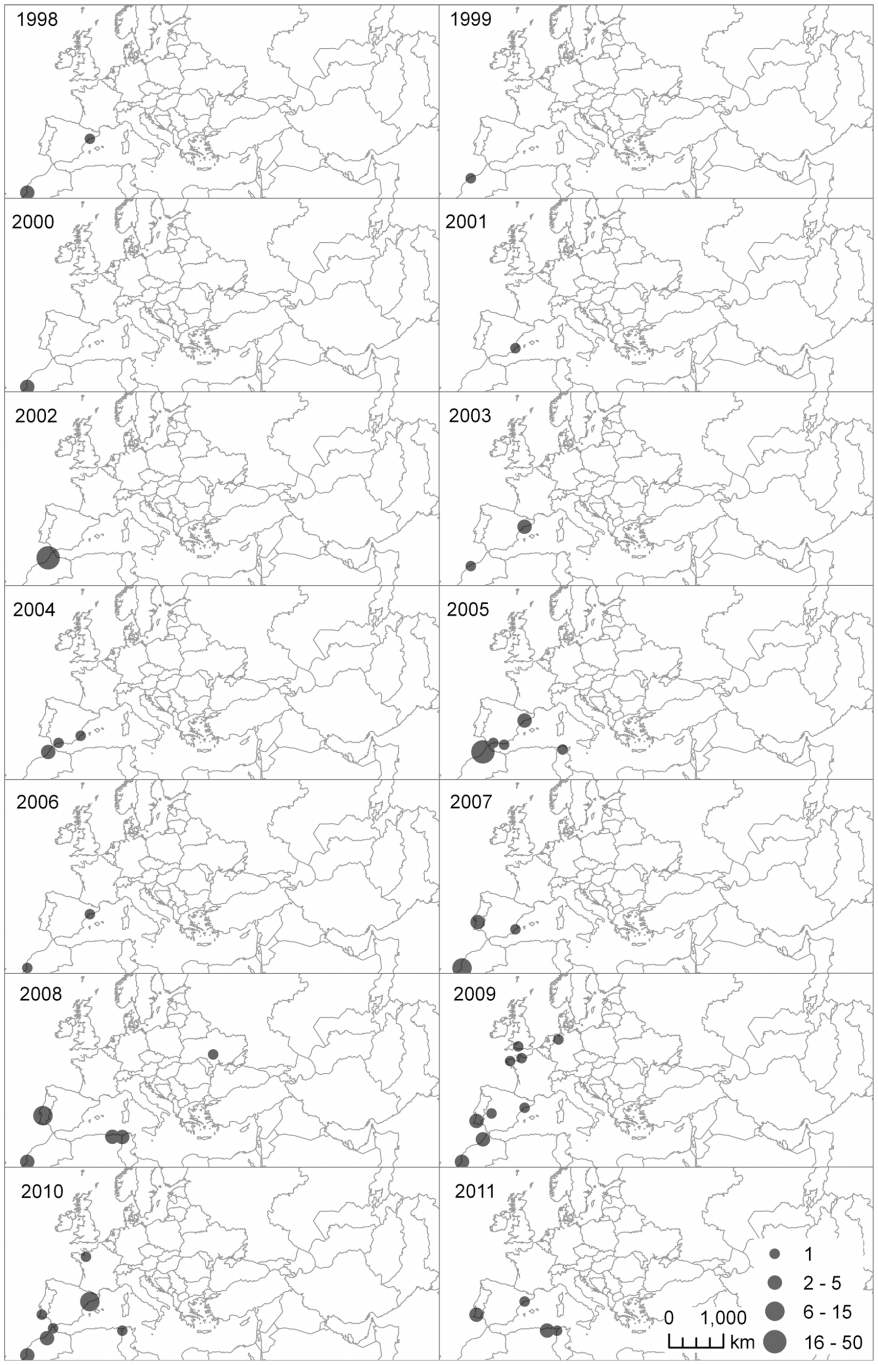
Range expansion according to resightings outside Doñana. Doñana-marked individuals resighted elsewhere for each year throughout the study period (from October to January, records from other months are not shown). Glossy ibises were seen in an increasing number of locations in parallel to the increase in the breeding population in Doñana. A sharp spreading of the population across Europe and northern Africa occurred during the 2005 drought. An observation from Barbados in September 2010 is not shown.

The growth of the Doñana population has been associated with an increasing number of observations and resighting locations outside Doñana. The spread to new and more distant areas sharply increased after the 2005 dry year (Figure 4). On average, considering all the autumns together, there was no difference in dispersal between sexes (Chi-square test 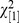 = 0.26, *n* females = 901, *n* males = 969, *P* = 0.6); nor was there for the period from 2005 to 2011 (i.e. since the last drought), (Chi-square test 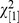 = 2.5, *n* females = 473, *n* males = 481, *P* = 0.11). On the other hand, a significantly higher proportion of males (16 of 19) than females (8 of 17) dispersed in the drought year 2005 (Chi-square test 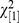 = 4, *P* = 0.04).

## Discussion

In this study we have found evidence that dispersal rates in a newly established population of glossy ibis changed over time as the population density increased. In a recent evaluation of the conservation state of the glossy ibis in Europe, it was considered of conservation concern in Europe, where it was in decline [41]. However, expansion across Europe and North-west Africa from Doñana is now well underway due to the good reproductive success in most years, transforming the conservation status of the species. However, our study demonstrates how this expansion is largely driven by droughts in Doñana.

### Population Dynamics

Several studies have focused on the effects of extreme events (like flooding or droughts) on survival and fecundity rates (reviewed in [42]). Nonetheless, other equally important parameters like dispersal or recruitment, crucial for understanding the population responses to extreme events, have scarcely been addressed [6]. Given current changes in climate, there is a critical need for further research on the effects of extreme environmental events on dispersal processes [5]. Droughts can prevent seasonal flooding of wetlands that are crucial for breeding of numerous waterbird species and therefore affect their survival and dispersal rates. Although we cannot rule out an effect on survival, this study demonstrates that glossy ibis dispersal was strongly enhanced by drought events.

We focused on factors affecting two different dispersal strategies: those of individuals present sporadically or regularly (we defined them as transients and residents respectively). Capture-resighting models were built to compute, for each time-interval, the probability of being a transient or dying just after the first resighting and, conditional on being still alive and in the area after the first interval, the probability of apparent dispersal (i.e. permanently emigrating from the area or dying). In other words, leaving apart the mortality contribution to these probabilities, we dealt with the probability of being a transient and with the emigration probability of residents. Multievent analyses indicated a clear positive effect of droughts on apparent dispersal. Resightings from beyond the study area and analysis on data collected simultaneously in Morocco and Doñana indicated that a drought event (2005) caused a dispersal peak. Moreover, we found strong support for a model relating the proportion of observations from outside with the probability of being transient over the study period. This suggests that apparent transience estimates are strongly related to transient dispersal probabilities over the whole study period, and not just for dry years. However, we did not find similar support for models of the apparent dispersal of residents.

Thus, decreasing apparent dispersal of residents over time (Figure 2) may potentially depend more on changes in true mortality rather than in dispersal propensity. However, we are not confident that this is the case, because illegal shooting at glossy ibis in rice fields and nearby roosting sites may have increased in the area in recent years (personal observation), so we would expect to find an increasing trend in apparent dispersal. On the other hand, it seems more plausible that residents decreased their dispersal propensity over time as they gained experience in the area [43], [44].

As a possible outcome of the “win-stay, lose-switch” strategy [45], individuals of any age may cue on their own breeding success and/or those of neighbours to make dispersal decisions [43], [46], [47]. Hence, breeding failure but also shortage of resources [48], [49] might explain why more individuals left the natal area in dry years. However, we did not find strong evidence for a correlation with the surface area of natural marshes that was used as a proxy for available resources (since no direct measure of resources was available). This may be because this is not a suitable proxy, since for other waterbirds there is evidence that extensive flooding and the associated high water depths lead to a reduction in resource accessibility [50]. Breeding failure owing to drought probably made more ibis individuals permanently emigrate from the area than in other years. In fact, in 2005 a new breeding colony settled in the Camargue wetlands (southern France), [51] and Portugal [52]. Since then several new breeding colonies have been discovered in North Morocco [53], Tunisia [54], Algeria [55] and northern France [56]. A number of Doñana-marked individuals have been observed in these new breeding sites, further suggesting that the Doñana population is fuelling the geographical expansion of the species.

Prospecting behavior in colonial species may serve to gather public information (e.g. breeding success) to help choose the breeding site in the next season [57]. Interestingly, in wet years, transient and resident ibis seemed to follow a different dispersal pattern. Firstly, transient propensity was possibly affected by breeding success at the colony. Although this effect was not significant according to the ANODEV procedure, it received support in terms of AICc in the final step of model selection (File S3; Table S1 in File S4). Several factors may reduce the power of the analysis, like: (*i*) limited time-series data, (*ii*) if most prospecting occurred in the late breeding season (as suggested by Boulinier et al. [57]), this effect may have been overlooked since we focused on individuals observed in the previous autumn, or (*iii*) conditions elsewhere also determine the probability of transients returning to Doñana. Furthermore, prospecting, inexperienced individuals of a colonial species like glossy ibis are more likely to depend on public information (e.g. breeding success) than residents, which are more likely to rely on their own experience (see [43], [58]). Secondly, unlike transients, residents decreased their propensity to disperse over time since the species became established in Doñana. The increasing experience of individuals might favor this process [43], [44]. Alternatively, different dispersal phenotypes may have a different fitness according to the time elapsed since colonization, favoring the prevalence of philopatric or disperser phenotypes respectively in recent and long-established populations [59]. Currently, we cannot establish the relative contribution (if any) of these two processes on the apparent increased philopatry of residents.

In general, juvenile birds are expected to be more prone to dispersal [45]. Nonetheless, we did not find a clear effect of age on dispersal propensity. This might be for several reasons: (*i*) under our modeling approach an age effect was tested only on transients (residents by definition were all adults), (*ii*) due to limited sample size we only tested for a difference between individuals one-year old and those of other ages and this may obscure any true correlation, and (*iii*) age might have opposite effects on true mortality and dispersal, counterbalancing each other.

The latter argument can also be applied for the unclear sex effect on apparent dispersal. Nonetheless, the autumn sex ratio of Doñana-born individuals in the natal area was initially strongly male-skewed but this gradually reversed and become female-skewed in the last two years (Figure 3). On the other hand, the sex ratio of chicks at the colony at the moment of marking was balanced except for the last two years when there were more males (unpublished results). Hence, even though more males apparently fledged in those last two years, more females were present during the autumn. This apparent contradiction might be explained if more males than females had dispersed in the last years, a possibility supported from the estimates of a model accounting for the interactive effect of sex and time variation on the apparent dispersal of residents (model 26: Table S2 in File S4; Figure S5 in File S6). We found significantly more males outside Doñana than inside in autumn 2005 (just after the last drought event), suggesting that males and females can use a different dispersal strategy according to other factors like environmental and demographic conditions. Also, since glossy ibis males are bigger than females [26], they might better withstand moderate levels of competition [60] causing a bias towards males at low density but not at high density when competition increased (Figure 3; Figure S5 in File S6).

Even though density has been found to affect dispersal in many studies [61], we did not find any support for a linear density effect on apparent transience or apparent dispersal of residents. The complexity of density effects with respect to other factors, as discussed above, may explain this result. On the other hand, this might be due to the population size being below a certain threshold above which density-dependent mechanisms start to be effective.

### Methodological Issues

Single-site capture-resighting models cannot distinguish between true mortality and permanent emigration from the area [62]. Ideally, to separately estimate dispersal and true mortality multi-site models [19] should be applied on longitudinal data collected from several locations covering most of the metapopulation range [20], [63]. Nonetheless, logistic and economic limitations often make this unfeasible and longitudinal data are typically available only from one single location where the population has been well monitored. To make multi-site modeling possible, pooling data at broad scales is a common practice but this can magnify survival and re-encounter heterogeneity, hence causing underestimation of sampling variance [64] and biased estimates [65]. Here, we used a single-site approach including specifically designed field-work and showed apparent dispersal mainly reflected dispersal variation [66]. It should be noted that we have only studied permanent emigration probabilities which refer to a one-way movement whereas movements in both direction are of interest. This represents a shortcoming of our single-site capture-resighting approach. However, our aim was to test hypotheses for the leaving stage of dispersal, not for the transfer and immigration stages [2], [36]. Neither did we aim to estimate absolute rates of dispersal or of true mortality.

On the other hand, temporary emigration by ibis is likely to have occurred throughout the study period. Markovian temporary emigration is known to affect the accuracy of transition estimates, but the effect of this bias tend to be small and non-existent when temporary emigration is random [67]. We do not consider that this is a major concern in our study since for testing hypotheses, the focus is on parameter time-variation, which does not require very accurate estimates.

## Conclusion

Extreme events are known to affect life-history traits like survival and reproduction [5]. The glossy ibis perfectly illustrates the effects of extreme drought on reproduction as a complete population skipped reproduction in Doñana during drought years. Furthermore, our findings highlight a causal effect between extreme events (like droughts) and dispersal which has been largely overlooked until now. Apart from a negative effect on reproduction, extreme drought episodes have fuelled the expansion of glossy ibis across the Mediterranean region, favoring the establishment of new colonies. These “catastrophic” episodes may be very important drivers in the population dynamics of highly mobile species such as colonial waterbirds. Further research is necessary to clarify the importance of such phenomena on the connectivity and metapopulation dynamics of waterbirds and other highly mobile species.

## Supporting Information

File S1 **Goodness-of-fit results.**
(DOC)

File S2 **Multievent probabilistic framework of the study.**
(DOC)

File S3 **Model selection procedure.**
(DOC)

File S4 **Supplementary tables.** Contains: **Table S1. Set of candidate models used to model average apparent dispersal estimates; Table S2. Age and sex effects on apparent dispersal probabilities; Table S3. Cohorts of marked individuals resighted at Doñana or in Morocco between 12th and 16th December 2010; Table S4. Set of candidate models from initial Event modeling (block 1 series 1); and Table S5. Set of candidate models from final Event modeling (block 1 series2).**
(DOC)

File S5 **Implementation in E-SURGE.**
(DOC)

File S6 **Supplementary figures.** Contains: **Figure S1. Apparent transience probabilities according to age and time; Figure S2. Resighting probabilities; Figure S3. Visual sexing probabilities for ring-marked chicks; Figure S4. Yearly probabilities of correct visual sexing for ringed-marked chicks; Figure S5. Apparent dispersal probabilities of residents according to sex.**
(DOC)
