# Supplementary material for: Environmental Instability as a Motor for Dispersal: A Case Study from a Growing Population of Glossy Ibis
Source: PLoS One. 2013 Dec 20;8(12):e82983. doi: 10.1371/journal.pone.0082983 (PMC3869753; doi:10.1371/journal.pone.0082983)
Supplement: File S1 — Goodness-of-fit results. (DOC) [file pone.0082983.s001.doc]

**GOF Results**

BEFORE SUPPRESSION OF FIRST ENCOUNTER:

Global TEST, number of groups =2 (Captured as juveniles, captured as adults)

df =92

Quadratic Chi2 =183.4267

->P-level=4.8508e-008

N(0,1) statistic for transient(>0) =6.6833

->P-level, two-sided test =2.3362e-011

->P-level, one-sided test for transience =1.1681e-011

N(0,1) signed statistic for trap-dependence =-2.7123

->P-level, two-sided test =0.0066813

TEST3.SR, group 1 = Captured as juveniles

N(0,1) statistic for transient(>0) =5.6269

P-level, two-sided test =1.8344e-008

P-level, one-sided test for transience =9.172e-009

*******************************************

TEST3.SR, group 2 = Captured as adults

N(0,1) statistic for transient(>0) =3.7834

P-level, two-sided test =0.00015467

P-level, one-sided test for transience =7.7337e-005

*******************************************

TEST2.CT, group 1 = Captured as juveniles

N(0,1) signed statistic for trap-dependence =-3.4311

trap-happiness<0 trap-shyness>0

P-level, two-sided test =0.00060114

*******************************************

TEST2.CT, group 2 = Captured as adults

N(0,1) signed statistic for trap-dependence =-0.15665

trap-happiness<0 trap-shyness>0

P-level, two-sided test =0.87552

*******************************************

AFTER SUPPRESSION OF FIRST ENCOUNTER:

Global TEST, number of groups =2 (Captured as juveniles, captured as adults)

df =68

Quadratic Chi2 =62.9805

->P-level=0.64948

N(0,1) statistic for transient(>0) =0.37264

->P-level, two-sided test =0.70941

->P-level, one-sided test for transience =0.35471

N(0,1) signed statistic for trap-dependence =-1.0815

->P-level, two-sided test =0.27949

TEST3.SR, group 1 = Captured as juveniles

N(0,1) statistic for transient(>0) =0.24878

P-level, two-sided test =0.80353

P-level, one-sided test for transience =0.40177

*******************************************

TEST3.SR, group 2 = Captured as adults

N(0,1) statistic for transient(>0) =0.28257

P-level, two-sided test =0.77751

P-level, one-sided test for transience =0.38876

*******************************************

TEST2.CT, group 1 = Captured as juveniles

N(0,1) signed statistic for trap-dependence =-1.6623

trap-happiness<0 trap-shyness>0

P-level, two-sided test =0.096452

*******************************************

TEST2.CT, group 2 = Captured as adults

N(0,1) signed statistic for trap-dependence =0.30148

trap-happiness<0 trap-shyness>0

P-level, two-sided test =0.76305
