# Supplementary material for: Environmental Instability as a Motor for Dispersal: A Case Study from a Growing Population of Glossy Ibis
Source: PLoS One. 2013 Dec 20;8(12):e82983. doi: 10.1371/journal.pone.0082983 (PMC3869753; doi:10.1371/journal.pone.0082983)
Supplement: File S2 — Multievent probabilistic framework of the study. (DOC) [file pone.0082983.s002.doc]

**Multievent probabilistic framework of the study**

Multievent models combine information from events with the underlying states to estimate probabilities of several parameters. A multievent model accounts for three parameter types: Initial State, State Transition and Event probabilities.

In this study we defined three underlying biological states:

✝ - Death or transient (referring to the state with zero probability of resighting due to its permanent emigration from the study area or to being dead)

♀ - Female alive at Doñana

♂ - Male alive at Doñana

The events, numbered as they appear in the data set, were four:

0 - Bird not resighted

1 - Bird resighted, visually identified as female when it was a chick

2 - Bird resighted, visually identified as male when it was a chick

3 - Bird resighted, not sexed visually when it was a chick

**INITIAL STATE**

This parameter refers to the probability that, when an individual is first resighted at Doñana in autumn, it is a male. Thus, assuming (*i*) there are no differences between p of resighting of males and females and (*ii*) the sampling scheme has not varied during the study period, this parameter is related to the sex ratio of the population [1, 2] of Doñana-born individuals at their natal site in autumn. By definition, the probability of being first captured as death or transient is zero. Therefore, the initial state probabilities are:

| ♀ | ♂ |
| --- | --- |
| 1-π | π |

π is the probability that, when an individual is first resighted at Doñana in autumn, it is a male.

**STATE TRANSITION**

This parameter type was divided in two steps. These probabilities are best represented in the form of stochastic matrices with departure states in rows and arrival states in columns. Transitions between genders were not allowed.

**Step 1 - Transience**; this step computes the probability that an individual first resighted on occasion *t* dies or permanently emigrates from the area in the interval between *t* and *t* + 1. As a consequence, it only applies for this interval (see below Additional file 5 for details on implementation in E-SURGE).

|  | ♀ | ♂ | ✝ |
| --- | --- | --- | --- |
| ♀ | 1-φ_f_ | 0 | φ_f_ |
| ♂ | 0 | 1-φ_m_ | φ_m_ |
| ✝ | 0 | 0 | 1 |

Where φ_f_ and φ_m_ refer respectively to the probability a female or a male has of permanently emigrating or dying in the interval after its first resighting.

**Step 2 - Residence (conditional on Transience)**; this step computes the probability a non-transient (i.e. one individual still available to be resighted after its first resighting) has of dying or permanently emigrating from the area between *t* and *t* + 1 (ψ_f_ for a female and ψ_f_ for a male).

|  | ♀ | ♂ | ✝ |
| --- | --- | --- | --- |
| ♀ | 1-ψ_f_ | 0 | ψ_f_ |
| ♂ | 0 | 1-ψ_m_ | ψ_m_ |
| ✝ | 0 | 0 | 1 |

The notation is the same used for the step 1.

**EVENT**

The Event probabilities relate the events to the underlying biological states. This parameter type has been divided into three steps.

**Step 1 - Resighting**; This estimates the probability (β_f_ and β_m_ for a female and male respectively) one individual has to be resighted at *t* + 1.

|  | not seen | live ♀ seen | live ♂seen |
| --- | --- | --- | --- |
| ♀ | 1 - β_f_ | β_f_ | 0 |
| ♂ | 1 - β_m_ | 0 | β_m_ |
| ✝ | 1 | 0 | 0 |

Same notation as above.

**Step 2 - Visual Sexing (conditional on Resighting)**; this estimates, conditional on being resighted, the probability (γ_f_ and γ_m_ for a female and male respectively) one individual has to be visually sexed when it was a chick.

|  | not seen | not sexed | live ♀ sexed | live ♂sexed |
| --- | --- | --- | --- | --- |
| not seen | 1 | 0 | 0 | 0 |
| live ♀ seen | 0 | 1 - γ_f_ | γ_f_ | 0 |
| live ♂seen | 0 | 1 - γ_m_ | 0 | γ_m_ |

Same notation as above

**Step 3 - Correctness (conditional on Resighting and Visual Sexing)**; This estimates, conditional on being resighted and visually sexed, the probability (δ_f_ and δ_m_ for a female and a male respectively) an individual has of being correctly visually sexed as a female or a male.

|  | 0 | 1 | 2 | 3 |
| --- | --- | --- | --- | --- |
| not seen | 1 | 0 | 0 | 0 |
| not sexed | 0 | 0 | 0 | 1 |
| live ♀ sexed | 0 | δ_f_ | 0 | 1 - δ_f_ |
| live ♂sexed | 0 | 0 | δ_m_ | 1 - δ_m_ |

Same notation as above

**References:**

1. Pradel R: **The stakes of Capture-Recapture Models with State Uncertainty**. In *Modeling Demographic Processes in Marked Populations*. Edited by Thomson DL, Cooch EG, Conroy MJ. New York: Springer; 2009:781–795.

2. Genovart M, Pradel R, Oro D: **Exploiting uncertain ecological fieldwork data with multi-event capture-recapture modelling: an example with bird sex assignment.** *Journal of animal ecology* 2012, **81**:970–977.
