# Supplementary material for: Environmental Instability as a Motor for Dispersal: A Case Study from a Growing Population of Glossy Ibis
Source: PLoS One. 2013 Dec 20;8(12):e82983. doi: 10.1371/journal.pone.0082983 (PMC3869753; doi:10.1371/journal.pone.0082983)
Supplement: File S3 — Model selection procedure. (DOC) [file pone.0082983.s003.doc]

**Model selection procedure**

Given the high number of biological parameters, we divided the analysis into two blocks, each one with two series: in block 1 we modeled Resighting, Visual sexing and Correctness, and in block 2 we modeled Initial State, Transience and Residence. In series 1 (Table 1, Table 2, Table 3 and Table S4) we modeled each biological parameter by keeping the others as general as possible and in series 2 (Table S1 and Table S5) we ran all the possible combinations of models which resulted to be within 2 ΔAICc in series 1. Thus, for each block we reached a supposed best model we named *bm1* (block 1, Table S5) and *bm2* (block 2, Table S1).

In block 1, we tested the trap-dependence effect on Resighting (sex effect was discarded by a preliminary analysis), and cohort and gender effects on Visual Sexing and Correctness. In the block 2, we tested several hypotheses concerning the possible effects of extrinsic factors on the sex ratio and both the apparent transience and residents apparent dispersal. All the tested covariates were standardized [1]. De-trended values of population abundance were used to test for density effects on residents apparent dispersal, since a significant linear effect (according to ANODEV) was found for this parameter type (details are given in the Methods section).

When modeling block 2, the structure of Event parameters was kept the same as for *bm1*, while the effects of age and gender on Transience and Residence were not tested. We specifically tested these effects on *bm2* (Table S2). The age effect was tested only on Transience because a non-transient individual apparent dispersal probability (hence modeled in Residence) was the same as for adults. Model averaging estimates [2] for Transition parameters were computed from all the models in block 2 series 2 (Table S1). All the other estimates proceed from *bm2*.

**References:**

1. Choquet R, Nogue E: *E-SURGE 1.8 User’s Manual*. Montpellier, UMR 5175, France: CEFE; 2011(September).

2. Burnham KP, Anderson DR: *Model Selection and Multimodel Inference: a Practical Information-theoretic Approach*. 2nd ed. New York: Springer-Verlag; 2002.
