# Supplementary material for: Environmental Instability as a Motor for Dispersal: A Case Study from a Growing Population of Glossy Ibis
Source: PLoS One. 2013 Dec 20;8(12):e82983. doi: 10.1371/journal.pone.0082983 (PMC3869753; doi:10.1371/journal.pone.0082983)
Supplement: File S4 — Supplementary tables. Contains: Table S1. Set of candidate models used to model average apparent dispersal estimates; Table S2. Age and sex effects on apparent dispersal probabilities; Table S3. Cohorts of marked individuals resighted at Doñana or in Morocco between 12th and 16th December 2010; Table S4. Set of candidate models from initial Event modeling (block 1 series 1); and Table S5. Set of candidate models from final Event modeling (block 1 series2). (DOC) [file pone.0082983.s004.doc]

**Table S1. The candidate set of models for block 2 series 2. We computed model averaged apparent dispersal estimates from this set of models. We ran all the combinations of effects found to be within 2 AICc units from the best model for each biological parameter (Transience, Residence and Initial State, see Table 1, 2 and 3). Model notation: as in Table 1, between parentheses are the effects considered on Initial State (IS), Transience (T) and R (Residence). The model in bold has the lowest AICc and hence represents the *bm2*.**

| Model | Model structure | *np* | Dev | AICc | ΔAICc | *wi* |
| --- | --- | --- | --- | --- | --- | --- |
| **22-2** | **IS[Trend] T[Dry years + *bs*] R[Dry years + Trend]** | **48** | **16064.61** | **16161.55** | **0.00** | **0.62** |
| 22-1 | IS[Trend] T[Dry years] R[Dry years + Trend] | 47 | 16068.71 | 16163.62 | 2.07 | 0.22 |
| 22-4 | IS[Trend] T[Dry years + *dens*] R[Dry years + Trend] | 48 | 16068.64 | 16165.58 | 4.03 | 0.08 |
| 22-3 | IS[Trend] T[Dry years + *fgm*] R[Dry years + Trend] | 48 | 16068.69 | 16165.63 | 4.08 | 0.08 |

**Table S2**. Multievent modeling of apparent transience and apparent dispersal of residents of Doñana glossy ibises related to age and sex effects. Age was tested only on apparent transience probability given that residents were all adults by definition. Model notation: as in Table S1, "T" refers to apparent transience and "R" refers to apparent dispersal of residents. Age was tested only as an addictive effect whereas sex was tested as an additive effect "Sex (+)", or as an interactive effect "Sex (*)".

| Model | Model structure | *np* | Dev | AICc | T ΔAICc | R ΔAICc | T*wi* | R*wi* |
| --- | --- | --- | --- | --- | --- | --- | --- | --- |
| 22-2 | No effect | 48 | 16064.61 | 16161.55 | 0.31 | 0.01 | 0.32 | 0.45 |
| 21 | Age and Sex (+) on Transients | 50 | 16062.02 | 16163.05 | 1.80 |  | 0.15 |  |
| 22 | Age on Transients | 49 | 16062.26 | 16161.24 | 0.00 |  | 0.37 |  |
| 23 | Sex (+) on Transients | 49 | 16064.29 | 16163.27 | 2.03 |  | 0.14 |  |
| 24 | Sex (*) on Transients | 51 | 16063.57 | 16166.63 | 5.39 |  | 0.03 |  |
| 25 | Sex (+) on Residents | 49 | 16062.56 | 16161.54 |  | 0.00 |  | 0.45 |
| 26 | Sex (*) on Residents | 51 | 16061.50 | 16164.56 |  | 3.02 |  | 0.10 |

**Table S3** Number of individuals from each cohort, resighted at Doñana or in Morocco between 12th and 16th December 2010.

| Site | Cohorts n | | | | | | | | | | |
| --- | --- | --- | --- | --- | --- | --- | --- | --- | --- | --- | --- |
|  | 2000 | 2001 | 2002 | 2003 | 2004 | 2006 | 2007 | 2008 | 2009 | 2010 |  |
| Doñana | 3 | 1 | 4 | 3 | 7 | 15 | 40 | 37 | 104 | 203 | 417 |
| Morocco | 1 | 7 | 12 | 17 | 58 | 14 | 33 | 45 | 91 | 138 | 416 |

**Table S4** Block 1 series 1 model selection. At this stage, the global model structure was held for all the Transition and Initial State parameters while the Event parameters were modeled one-by-one by keeping the other two fixed as from the global model. Hence, models 1_1 to 1_3 aimed to find the best structure for Resighting, models 1_1, 1_4, 1_5 for Visual Sexing, and models 1_1, 1_6, 1_7 for Correctness. Model notation: R, Resighting (parameter type); VS, Visual Sexing; C, Correctness; trap.tt, trap effect with individuals resighted at the next session from their first resighting having a time-varying p of encounter different from the p of being resighted at subsequent sessions (which was also time-varying); trap.t+t, the same as trap.tt but the p values at subsequent sessions were additively time-varying with respect to the p values at the second session from the first resighting; t, time varying; sex, sex effect; cohort, birth-year effect. Irrelevant models such as those assuming constant time effect on Resighting are not reported here.

| Model | Structure | np | Deviance | ΔICc | R - ΔAICc | VS - ΔAICc | C - ΔAICc |
| --- | --- | --- | --- | --- | --- | --- | --- |
| 11-1 | R {trap.tt} VS{sex+cohort} C{sex+cohort} | 96 | 15997.98 | 16193.75 | 9.56 | 0.00 | 0.85 |
| 11-2 | R{trap.t+t} VS{sex+cohort} C{sex+cohort} | 85 | 16011.35 | 16184.31 | 0.12 |  |  |
| 11-3 | R{t} VS{sex+cohort} C{sex+cohort} | 84 | 16013.30 | 16184.19 | 0.00 |  |  |
| 11-4 | R{trap.tt} VS{sex} C{sex+cohort} | 83 | 17110.70 | 17279.51 |  | 1085.77 |  |
| 11-5 | R{trap.tt} VS{cohort} C{sex+cohort} | 95 | 16023.07 | 16216.76 |  | 23.01 |  |
| 11-6 | R{trap.tt} VS{sex+cohort} C{sex} | 85 | 16106.24 | 16279.19 |  |  | 86.30 |
| 11-7 | R{trap.tt} VS{sex+cohort} C{cohort} | 95 | 15999.21 | 16192.90 |  |  | 0.00 |

**Table S5** Block 1 series 2 model selection. At this stage we run all the models arising from all the combinations of effects found to be within 2 ΔAICc from the best model for each parameter type modeled in Block 1 series 1. The Event structure as from the lowest AICc model found (*bm1*) at this stage was retained for modeling in Block 2. Model notation as from Table S4.

| Model | Structure | np | Deviance | AICc | ΔAICc |
| --- | --- | --- | --- | --- | --- |
| 12-1 (*bm1*) | R{t} VS{sex+cohort} C{cohort} | 83 | 16014.545 | 16183.3603 | 0 |
| 12-2 | R{t} VS{sex+cohort} C{sex+cohort} | 84 | 16013.3019 | 16184.1856 | 0.8253 |
| 12-3 | R{trap.t+t} VS{sex+cohort} C{cohort} | 84 | 16012.5737 | 16183.4574 | 0.0971 |
| 12-4 | R{trap.t+t} VS{sex+cohort} C{sex+cohort} | 85 | 16011.3322 | 16184.2851 | 0.9248 |
