# Supplementary material for: Environmental Instability as a Motor for Dispersal: A Case Study from a Growing Population of Glossy Ibis
Source: PLoS One. 2013 Dec 20;8(12):e82983. doi: 10.1371/journal.pone.0082983 (PMC3869753; doi:10.1371/journal.pone.0082983)
Supplement: File S5 — Implementation in E-SURGE. (DOC) [file pone.0082983.s005.doc]

**Implementation in E-SURGE**

The data set was prepared according to the "HEADED format" [1]. Once E-SURGE was launched, the data set was loaded and divided into covariate-groups according to (*i*) molecular sex, (*ii*) age at first resighting, and (*iii*) cohort. Thus, 84 groups were defined according to birth-year (14 cohorts), age (juvenile or adult) at first resighting and molecular sex (female, male or unknown).

Then, in "Data Input / Modify" the number of states were reduced from four to three, the number of events was left equal to four and the number of age classes was reduced to two (instead of 13 available).

In "Advanced Numerical / Modify", to improve results precision and facilitate convergence, we diminished the tolerance to parameter change to 1e-008 [1]. To reduce possibility of convergence on a false (local) minimum, we set "Advanced Numerical / Initial Values" to Multiple Random (= 5).

Afterwards, in "COMPUTE A", in this order we:

1) Loaded the GEPAT structure:

%%%% VERSION 2.0 %%%%%%

3

%%%% Initial state %%%%%%

1

1 2 IS

* P

%%%% Transition %%%%%%

2

3 3 Transience

* - p

- * p

- - *

3 3 Residence

* - p

- * p

- - *

%%%% Event %%%%%%

3

3 3 Resighting

* b -

* - b

* - -

3 4 Visual Sexing

* - - -

- * p -

- * - p

4 4 Correctness

* - - -

- - - *

- p * -

- * p -

2) Defined the GEMACO structure for the current model. The GEMACO structure of the global model was:

For Initial State: IS - Step 1 - (16): fem+mal+unk.t

For Transition: Transience - Step 1: a(1).[edad+f+t]+a(2)

For Transition: Residence - Step 2: f+t

For Event: Resighting - Step 1: firste+[nexte.[captjuv.a(2)].t]+[nexte.[[captjuv.a(3)]&captad].t]

For Event: Visual Sexing - Step 2: birthyear+f

For Event: Correctness - Step 3: f+birthyear

Where these shortcuts were used:

"fem" = "molecularly sexed as females",

"mal" = "molecularly sexed as males",

"unk" = "molecularly unsexed ",

"edad" = "first resighting as juvenile vs first resighting as adult",

"captjuv" = "first time resighted being a juvenile",

"captad" = "first time resighted being an adult",

"birthyear" = "birth-year"

3) Set the IVFV:

For the Initial State, the probability of "fem" to be initially resighted as a male was fixed to zero and that of "mal" to one. For the "Transition - Step 1" the last parameter, corresponding to a(2) was set to zero since Transience by definition must apply only to the interval after first resighting. For "Event - Step 1", as usual, the first parameter was set to one because it refers to the probability of resighting for the first resighting of any individual.

4) RUN

**References:**

1. Choquet R, Nogue E: *E-SURGE 1.8 User’s Manual*. Montpellier, UMR 5175, France: CEFE; 2011(September).
