# Supplementary material for: Environmental Instability as a Motor for Dispersal: A Case Study from a Growing Population of Glossy Ibis
Source: PLoS One. 2013 Dec 20;8(12):e82983. doi: 10.1371/journal.pone.0082983 (PMC3869753; doi:10.1371/journal.pone.0082983)
Supplement: File S6 — Supplementary figures. Contains: Figure S1. Apparent transience probabilities according to age and time; Figure S2. Resighting probabilities; Figure S3. Visual sexing probabilities for ring-marked chicks; Figure S4. Yearly probabilities of correct visual sexing for ringed-marked chicks; Figure S5. Apparent dispersal probabilities of residents according to sex. (DOC) [file pone.0082983.s006.doc]

**Figures**

**Figure S1**. Apparent transience probability of Doñana-born glossy ibises resighted for the first time as juveniles (triangles, short-dashed line) and as adults (squares, long-dashed line). Estimates (95%CI) are from model 22 (Table S2).

**Figure S2.** Probability of resighting at Doñana of native glossy ibises in autumn (October - December). Estimates (95%CI) are from the model with the lowest AICc (*bm2* in Table S1).

**Figure S3.** Probability for chicks of being visually sexed depending on birth year (best model for Visual Sexing, cfr Table S4 and Table S5). Females (short-dashed line) were more likely to be visually sexed than were males (long-dashed line). Estimates (95%CI) are from the model with the lowest AICc (22-2).

**Figure S4.** Probability for chicks of being correctly sexed visually in each year (best model for Correctness, cfr Table S4 and Table S5). Visual sexing was a quite reliable method but its reliability varied according to years. Estimates (95%CI) are from the model with the lowest AICc (22-2).

**Figure S5.** Residents' apparent dispersal probability of females (triangles, short-dashed line) and males (squares, long-dashed line). Estimates (95%CI) are from model 26 (Table S2).


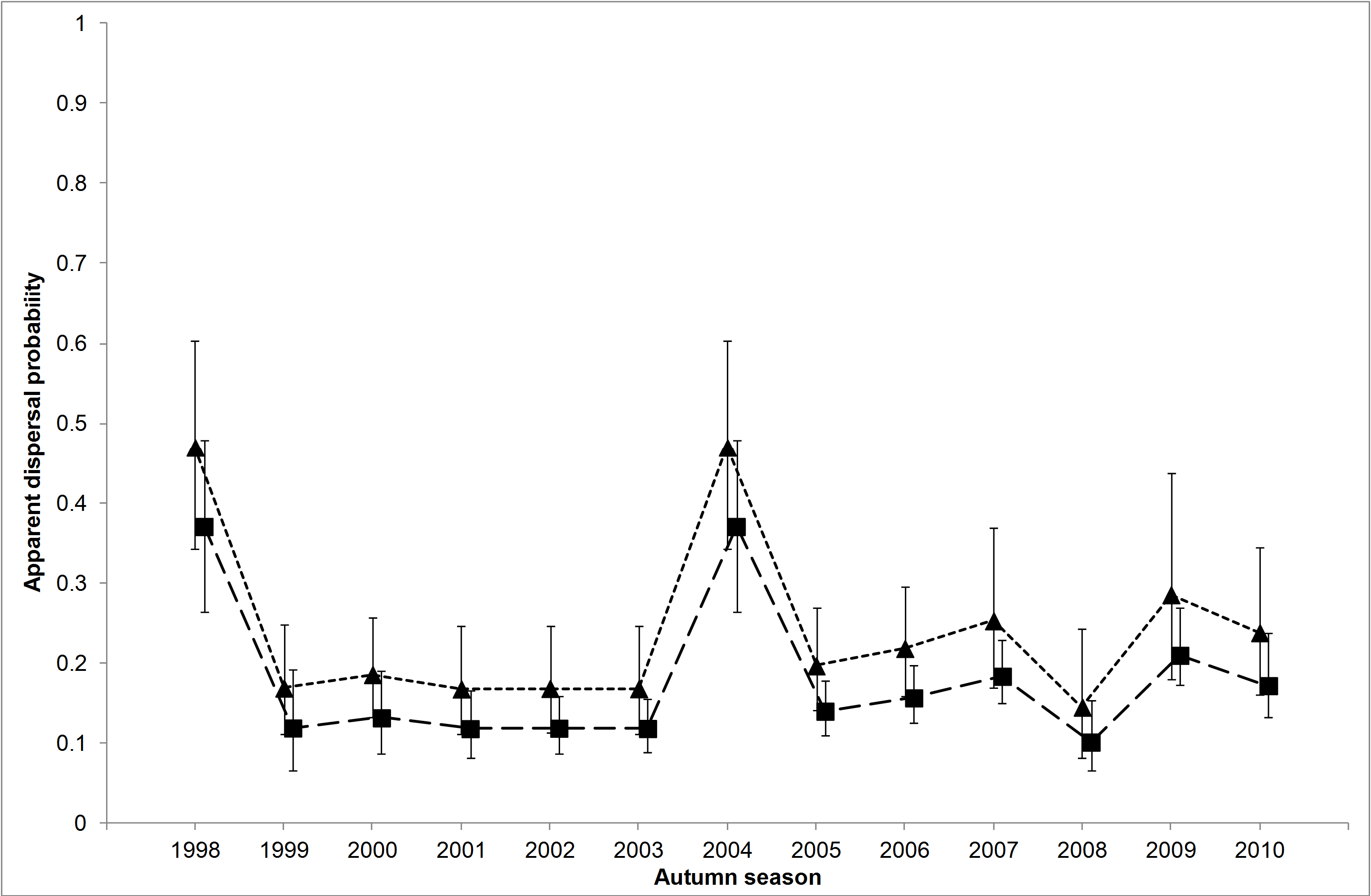


Fig. S1


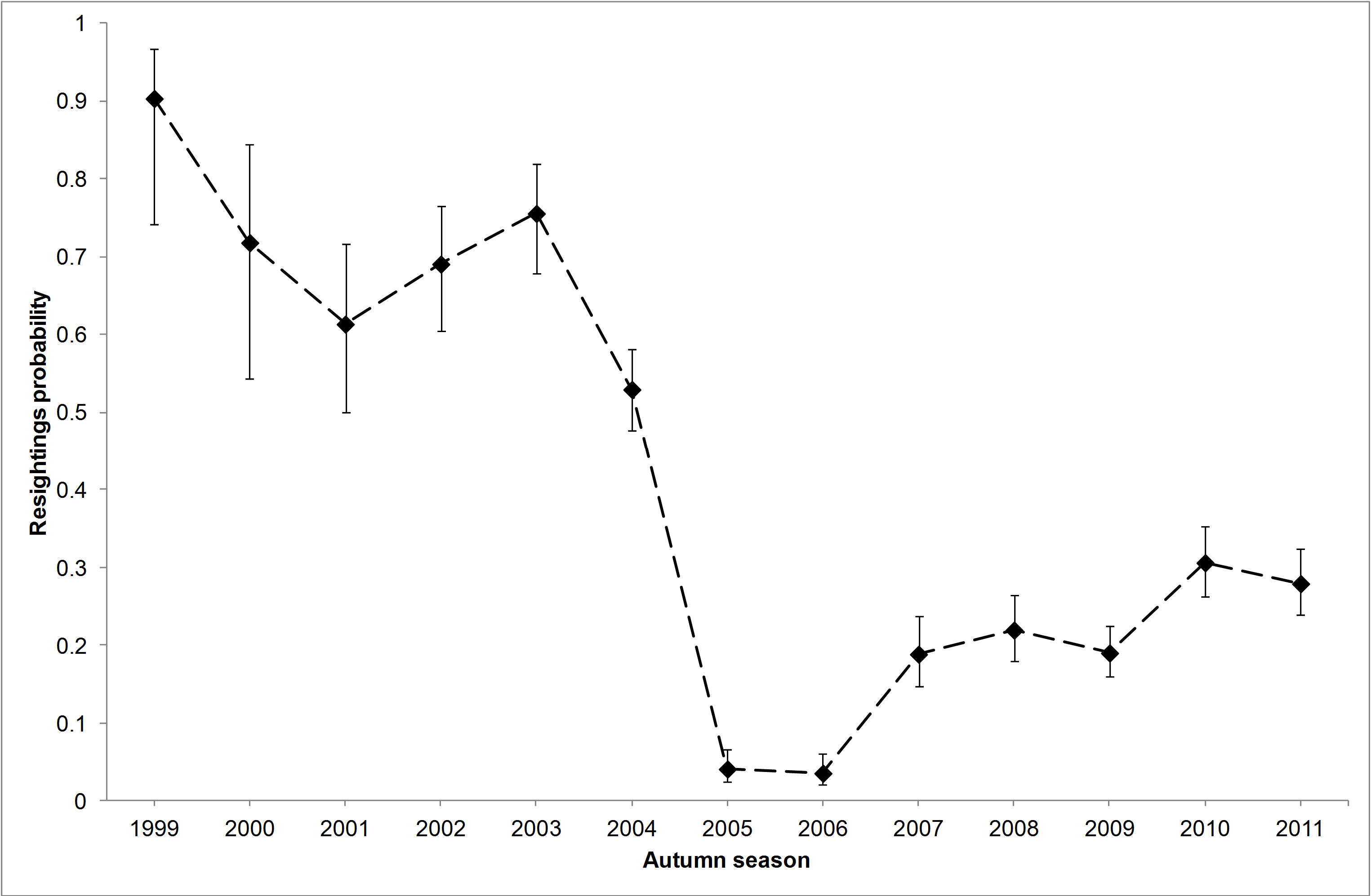


Fig. S2


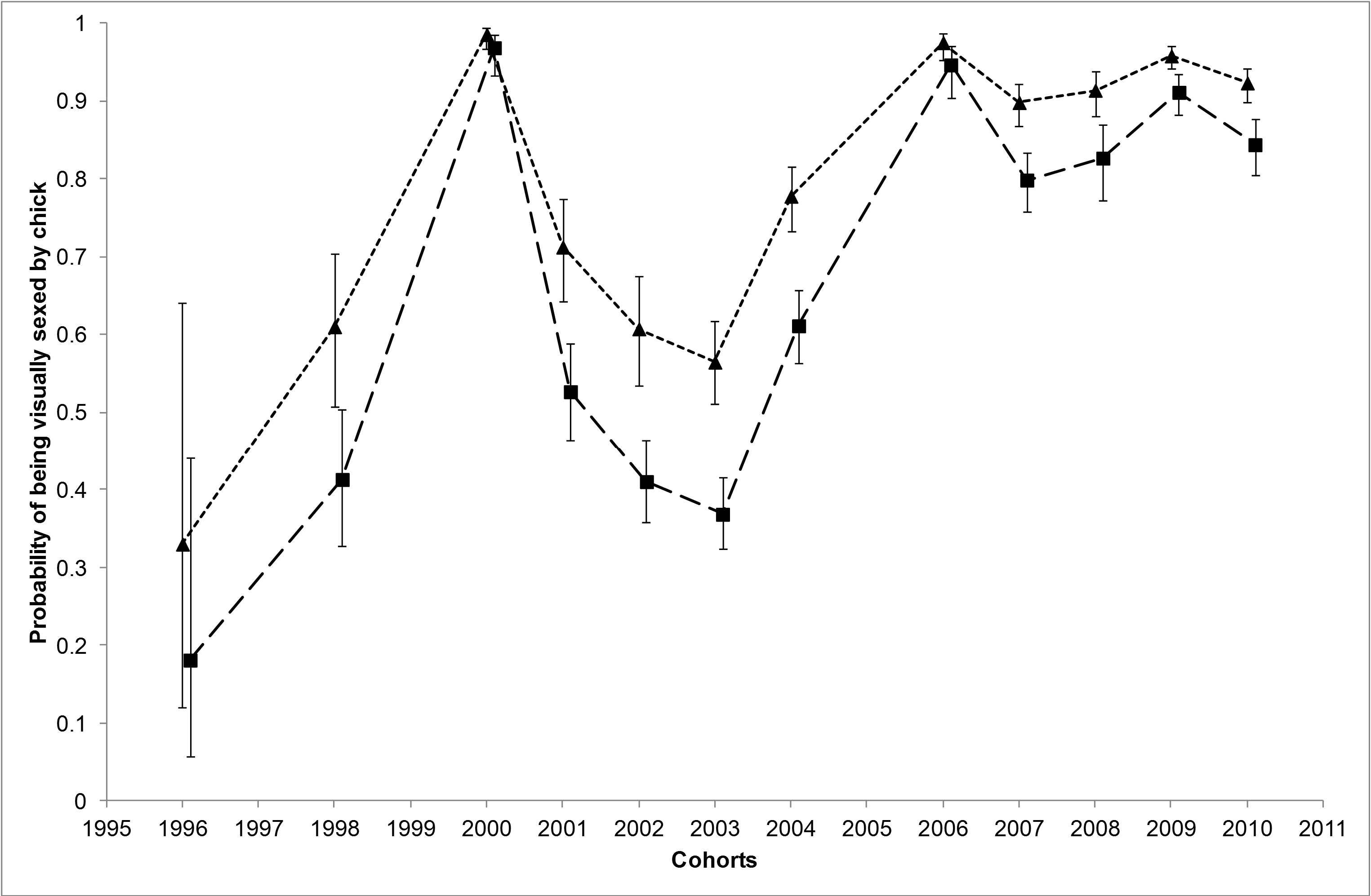


Fig. S3


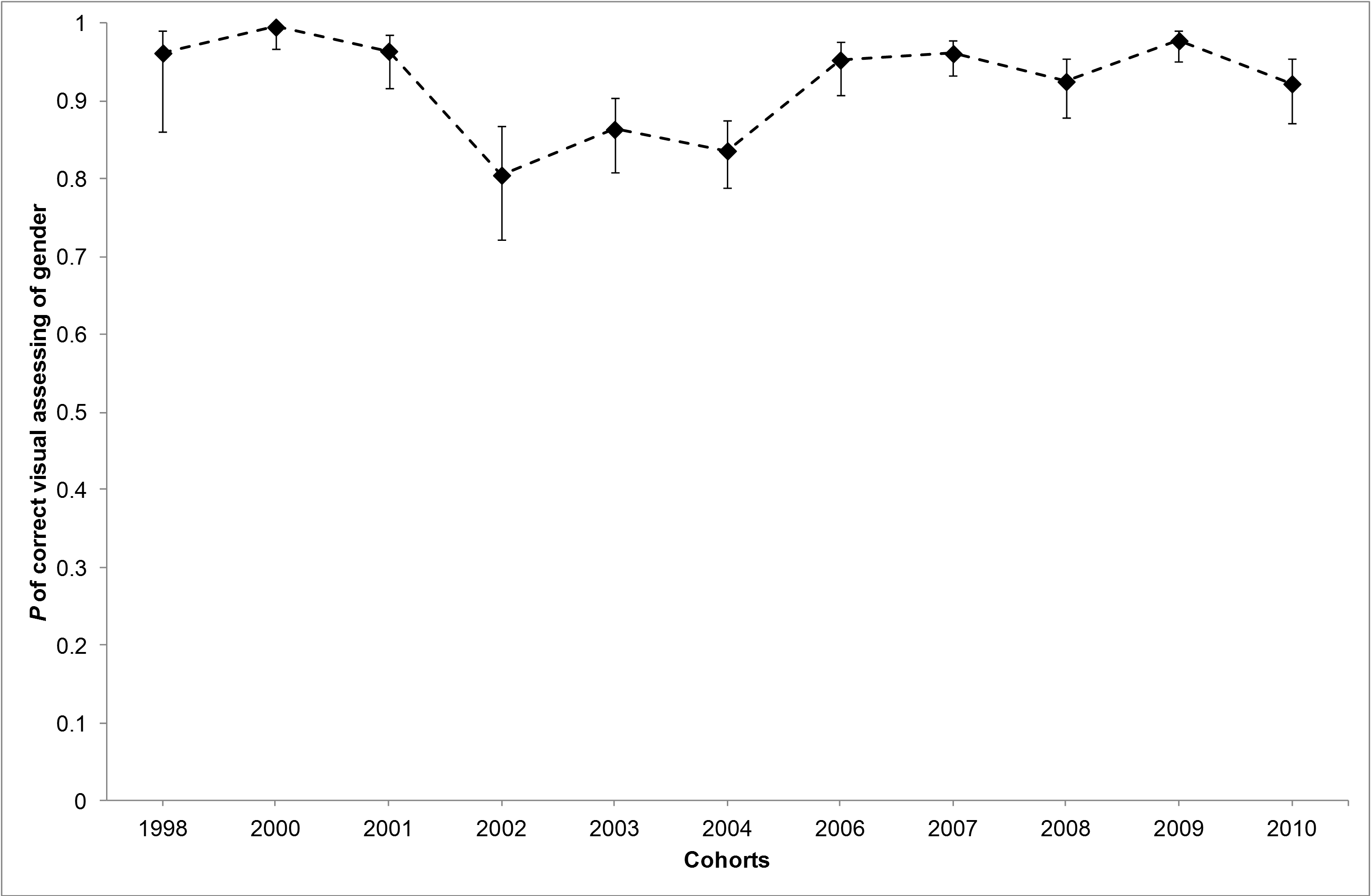


Fig. S4


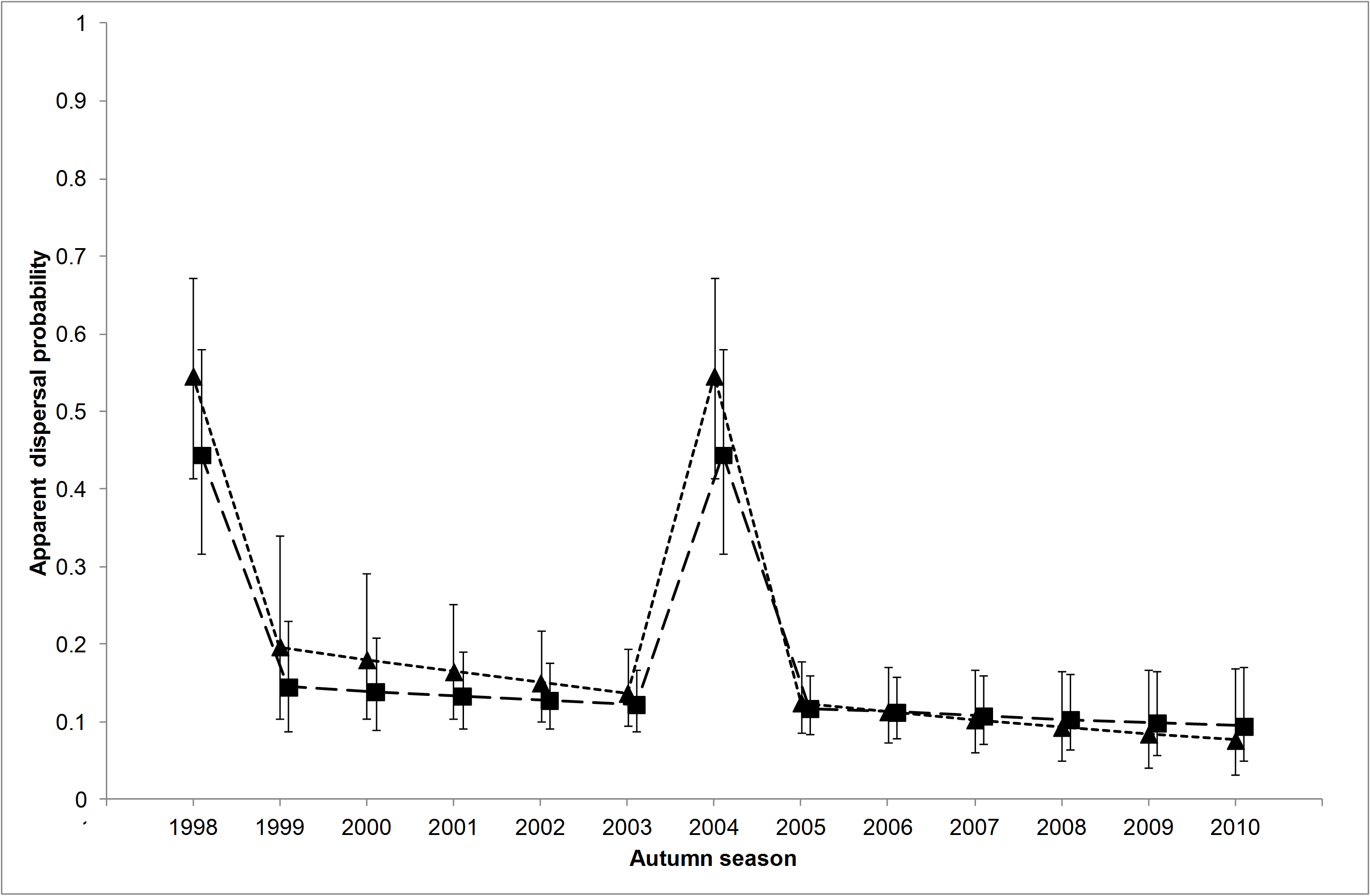


Fig. S5
